# Clinical challenges in the era of multiple and extensively drug-resistant tuberculosis

**DOI:** 10.26633/RPSP.2017.167

**Published:** 2017-12-26

**Authors:** Rafael Laniado-Laborín

**Affiliations:** 1 Facultad de Psicología y Medicina Universidad Autónoma de Baja California TijuanaBaja California Mexico Facultad de Psicología y Medicina, Universidad Autónoma de Baja California, Tijuana, Baja California, Mexico.

**Keywords:** Tuberculosis, multidrug-resistant tuberculosis, latent tuberculosis, diagnosis, therapeutics, social determinants of health, Tuberculosis, tuberculosis resistente a múltiples medicamentos, tuberculosis latente, diagnóstico, terapéutica, determinantes sociales de la salud, Tuberculose, tuberculose resistente a múltiplos medicamentos, tuberculose latente, diagnóstico, terapêutica, determinantes sociais da saúde

## Abstract

*In 2014, there were 480 000 new cases of multidrug-resistant tuberculosis (MDR-TB) around the world, but only 25% of them were diagnosed and reported. Drug resistance in TB is necessarily a laboratory diagnosis. An urgent priority in everyday practice is to diagnose tuberculosis and rule out drug resistance as quickly and as accurately as possible. However, worldwide, only 12% of new bacteriologically confirmed TB cases and 58% of previously treated TB cases were tested for drug resistance in 2014*.

*New tools for diagnosis of TB and drug-resistant TB have been introduced for clinical practice during the past decade. Those new tools can detect and identify drug resistance to antituberculosis drugs in less than 24 hours, and they should be urgently integrated into clinical practice, especially in high-burden regions*.

*Ongoing transmission of TB generates new infections, and this infected population is the inexhaustible source of new TB cases. If we are really determined to stop the global TB epidemic, we need to treat active cases and also halt the transmission of the infection. The only strategy for preventing the development of active disease in individuals with subclinical infection is to give treatment for this latent infection*.

*Global control of TB requires a huge investment of funds to address current detection and treatment gaps. We must reconsider our current strategy and combine social components with biomedical interventions. This will require the development of alliances between government and civil society, as well as leadership and true political commitment at the highest level of government*.

## RESUMEN

En el 2014 se presentaron 480 000 nuevos casos de tuberculosis multirresistente, pero solo se diagnosticó y notificó 25% de ellos. La farmacorresistencia en la tuberculosis se diagnostica necesariamente por medio de pruebas de laboratorio. En la práctica clínica diaria resulta urgente y prioritario poder diagnosticar la tuberculosis y descartar la farmacorresistencia con la mayor rapidez y exactitud posibles. Sin embargo, en todo el mundo, apenas 12% de los nuevos casos de tuberculosis bacteriológicamente confirmados y 58% de los casos ya tratados se sometieron a prueba de farmacorresistencia en el 2014.

En los diez últimos años se han dado a conocer nuevas herramientas para el diagnóstico de la tuberculosis y la tuberculosis farmacorresistente en la práctica clínica. Esas herramientas nuevas permiten detectar e identificar la resistencia a medicamentos antituberculosos en menos de 24 horas, por lo que deberían integrarse urgentemente a la práctica clínica, especialmente en las regiones con una carga de enfermedad alta.

La persistencia de la transmisión de la tuberculosis genera nuevas infecciones, y la población infectada es una fuente inagotable de nuevos casos de esta enfermedad. Si estamos realmente decididos a poner fin a la epidemia mundial de la tuberculosis, tenemos que tratar los casos activos y también detener la transmisión de la infección. La única estrategia para prevenir la aparición de la enfermedad activa en personas con infección subclínica es administrar tratamiento contra esta infección latente.

El control mundial de la tuberculosis requiere una enorme inversión de fondos para cerrar las brechas existentes en la detección y el tratamiento. Debemos reconsiderar nuestra estrategia actual y combinar los componentes sociales con las intervenciones biomédicas. Esto obliga a conformar alianzas entre el gobierno y la sociedad civil, y requiere del liderazgo y de un verdadero compromiso político de las más altas instancias gubernamentales.

## RESUMO

Em 2014, houve 480 mil novos casos de tuberculose (TB) resistente a múltiplos medicamentos, porém apenas 25% foram diagnosticados e notificados. A resistência aos medicamentos na TB requer necessariamente que seja feito um diagnóstico laboratorial. É prioridade na prática clínica diária diagnosticar a TB e descartar a resistência aos medicamentos o mais rápido e com maior precisão possível. Porém, em 2014, o teste da resistência aos medicamentos foi realizado mundialmente em apenas 12% dos novos casos de TB com confirmação bacteriológica e em 58% dos casos de TB com tratamento anterior.

Novas ferramentas para o diagnóstico de TB e TB resistente a múltiplos a medicamentos foram introduzidas na prática clínica na última década. São ferramentas com capacidade de detectar e identificar a resistênciaaos medicamentos antituberculose em menos de 24 horas e, portanto, é imprescindível que sejam integradas à prática clínica, sobretudo em regiões de elevada carga da doença.

A transmissão contínua da TB causa novas infecções, sendo a população infectada uma fonte inesgotável de novos casos da doença. Se estivermos realmente determinados a conter a epidemia global de TB, é preciso tratar os casos ativos e interromper a transmissão da infecção. A única estratégia para prevenir o surgimento de doença ativa em indivíduos com infecção subclínica é o tratamento da infecção latente.

O controle global da TB requer um enorme investimento financeiro para sanar as falhas atuais de detecção e tratamento da doença. A estratégia atual deve ser reexaminada e combinar componentes sociais e intervenções biomédicas. Faz-se necessário forjar alianças entre o governo e a sociedade civil bem como assumir a liderança e o firme compromisso no mais alto nível político.

The ability of *Mycobacterium tuberculosis* (MTB) to acquire mutations that result in resistance to antituberculosis drugs was described when the very first clinical trial with streptomycin was conducted 70 years ago (1). Unfortunately, we have allowed the bacillus to mutate to a practically untreatable form through the selective pressure of inadequate treatment regimens administered by national tuberculosis (TB) programs that are poorly structured and implemented (2).

Worldwide resistance to antituberculosis drugs is preventing the control and eventual eradication of TB, which is still a leading cause of death globally (3). The World Health Organization (WHO) estimated that globally in 2015 there were 580 000 new cases of multidrug-resistant TB (MDR-TB, that is, with simultaneous resistance *to at least* rifampin and isoniazid) or rifampin-resistant TB (RR-TB), but only 125 000 of them (20%) were diagnosed and reported. It is also estimated that 250 000 patients died from MDR-TB/RR-TB in 2015. According to the WHO 2016 Global Tuberculosis Report (4), in 2015, 3.9% of new TB cases and 21% of previously treated cases had MDR-TB/RR-TB; approximately 9.5% of those had extensively drug-resistant TB (XDR-TB, that is, with resistance to *at least* rifampin, isoniazid, any fluoroquinolone, and any second-line injectables).

Even more serious, XDR-TB strains have now mutated to what has been called totally drug-resistant TB (TDR-TB), which represents the most extreme form of drug resistance. Patients with TDR-TB harbor strains that are resistant to all the drugs for which we have the ability to test in a mycobacterial laboratory. The WHO has recommended avoiding the use of the TDR-TB term since these patients still might have one or two “usable drugs” available for treatment. The term recommended by the WHO for these patients is resistance beyond extensively drug-resistant tuberculosis (2).

Highly resistant MTB strains (MDR, XDR, and beyond XDR) will be transmitted in the community, leading to treatment failure when these new cases are treated empirically with a combined regimen of the first-line drugs (5).

MDR-TB and XDR-TB require treatment with drugs that are generally less effective, more toxic, and considerably more expensive than first-line drugs; in addition, treatment must be prolonged for 18 to 24 months. This reduced efficacy and increased costs of drugregimens make the treatment of MDR-TB/XDR-TB virtually unaffordable to TB programs in countries with limited resources, which, almost without exception, have the highest rates of drug-resistant TB (6).

This article will discuss current challenges in the prevention, diagnosis, and treatment of drug-resistant TB, as well as some of the possible solutions to these challenges.

## CHALLENGES IN DIAGNOSING TUBERCULOSIS

Drug resistance in TB is necessarily a laboratory diagnosis since there are no clinical signs or symptoms that can be used to diagnose a case caused by a drug-resistant MTB strain.

According to the WHO 2016 report (4), globally, only 57% of the pulmonary cases reported to WHO were bacteriologically confirmed. The undiagnosed cases, including ones caused by drug-resistant strains, will go untreated and will transmit the disease in the community. An urgent priority is to diagnose all cases as early as possible, including those that are drug resistant, and treat them until they are cured.

Therefore, an imperative in everyday practice is to diagnose tuberculosis and rule out drug resistance as quickly and as accurately as possible. This will require an increase in laboratory capacity *in every country* in order to allow the detection of drug resistance to first- and second-line drugs *in every case* of TB. In fact, one of the core components of the WHO post-2015 End TB Strategy is *universal* drug susceptibility testing (DST) (4).

Sputum microscopy is usually the first diagnostic test carried out to detect TB. It is rapid, widely available, quite specific, and very low cost, thus affordable in high-burden countries. Microscopy, however, cannot distinguish between drug-susceptible and drug-resistant mycobacteria. Therefore, that test should be followed by rapid tests to detect drug resistance. New cases that have been infected in the community with a drug-resistant strain and are treated empirically with a standardized first-line drugs regimen will frequently fail, amplify resistance, and prolong the chain of transmission (7).

The traditional diagnosis strategy for drug-resistant TB is phenotypic culture-based DST. The most important limitation of this strategy is its slow turnaround time; culture DST on a clinical isolate can take up to 16 weeks (4). However, a greater problem is that in most high-burden countries there are very few (and in many countries there are no) reference laboratories. In addition, the infrastructure for sample transportation to centralized or reference laboratories located outside the country is often suboptimal (8).

Fortunately, new tools for diagnosis of tuberculosis and drug-resistant tuberculosis have been introduced for clinical practice over the past decade. Some of these tests have received WHO endorsement and are now being used routinely in some countries (7). These include the Xpert MTB/RIF (Cepheid, Sunnyvale,California, United States of America) and such line probe assays (LPAs) as the GenoType MTBDR*plus* VER 2.0 and the GenoType MTBDR*sl* VER 2.0 (Hain Lifescience, Nehren, Tübingen, Germany). The Xpert MTB/RIF can identify *M. tuberculosis* and detect resistance to rifampin in just 2 hours, while the LPAs provide identification and can detect mutations associated with resistance to isoniazid, rifampin, fluoroquinolones, and second-line injectables in 24 hours (9).

The Xpert MTB/RIF is an automated, cartridge-based nucleic amplification assay. One of its advantages is that it can be used at the point of care since it does not require a sophisticated molecular biology laboratory or highly trained personnel to deliver molecular results. The main disadvantage of current Xpert equipment is that it requires electricity, and that the cartridges must be maintained at 28 °C or lower.

The LPAs’ main advantage is that they can diagnose MDR-TB/XDR-TB in just 24 hours. An important drawback is that they require a specialized laboratory and highly trained personnel, representing large investments that many countries are unable or unwilling to pay for. In practice, this means this technology remains largely limited to centralized reference laboratories (9).

Advances in whole-genome sequencing (WGS) have reduced the cost of sequencing the whole mycobacterium genome, and also increased the speed of sequencing, with the potential for cutting the turnaround time from weeks to just a few hours. As soon as the liquid culture system reports growth (7-10 days), WGS could be carried out directly from the liquid culture. This would allow for the identification of the precise subspecies and also detect resistance to all remaining drugs. Currently, WGS still cannot replace phenotypic DST for all antituberculosis drugs, given the incomplete understanding of the genetic basis of drug resistance (10).

In response to this challenge, high-burden countries must upgrade and streamline their laboratory networks. Molecular techniques should replace phenotypic methods for initial diagnosis and detection of drug resistance, while traditional cultures will still be needed during follow-up to detect viable bacilli. The Xpert system should be introduced at point-of-care facilities for rapid detection of rifampin resistance, and LPAs can diagnose MDR-TB and XDR-TB in just a couple of days, allowing for the early institution of effective treatment.

## CHALLENGES IN TREATING DRUGRESISTANT TUBERCULOSIS

Even with appropriate treatment regimens, success rates for MDR-TB range between 62% and 69% (11); the success rate for XDR-TB treatment is even lower, just 40%. According to the currently reported success rates, the probability of a cure for a patient with XDR-TB plus resistance to all second-line drug injectables is practically the same now as was the probability of cure for patients with TB in the pre-antibiotic era (12).

Only a minority of MDR-TB patients are being treated with an optimal regimen. Given that fact, a massive scale-up in diagnosis and treatment is needed to stop the ongoing transmission and the ever-growing proportion of drug-resistant cases (6). If these patterns are not changed, the exorbitant costs of treating MDR-TB and XDR-TB will become unaffordable for low-income countries (13).

## CONTROLLING AND EVENTUALLY ELIMINATING TUBERCULOSIS

As described below, there are three major problems that must be addressed if we want to control and someday eliminate TB.

### Disease transmission

Ongoing transmission of tuberculosis generates new infections, and this infected population is the inexhaustible source of new TB cases. If we are really determined to stop the global TB epidemic, we must treat active cases and also halt the transmission of the infection. This will require stepping up from passive case-finding to targeted active case-finding. That means actively screening individuals at increased risk of having the disease, diagnosing them, and putting them under treatment as soon as possible to render them noninfectious, and, as a result, stopping the chain of transmission. The passive case-finding approach delays the diagnosis and prolongs the infectious period of a case, with the inevitable transmission among family members and the community by the time the case is detected and put under treatment.

### Treatment of latent infection

Identifying infected individuals who could progress to active (and infectious) disease and treating their subclinical infection is a central strategy for interrupting TB transmission, reducing the burden of the disease, and moving toward global elimination.

The only strategy for preventing the development of active disease in individuals with subclinical infection is to give treatment for this latent infection.

A direct measurement tool for *M. tuberculosis* infection in humans is currently unavailable, so there is no gold standard for the diagnosis of latent tuberculosis infection (LTBI). The tuberculin skin test (TST) and interferon-gamma release assays (IGRAs) indirectly measure TB infection by detecting the memory T-cell response that signifies the presence of host sensitization to *M. tuberculosis* antigens (14).

While treatment of latent infection is a vital component of the TB elimination strategy, it is unfortunately the most underutilized option of all the proven tools available for the global control of TB. Virtually all high-burden countries have focused almost exclusively on the detection and treatment of active cases. Although most of them have guidelines and policies recommending treatment of latent infection, implementationof such recommendations is virtually nil. One of the barriers for putting it into practice is the duration of the most frequently recommended therapy, isoniazid for nine months.

To maximize the impact on the global TB epidemic, preventive therapy must be implemented in conjunction with the tracking of case contacts and other high risk individuals (e.g., people with HIV infection, recipients of tumor necrosis factor alpha blockers, and people with diabetes); targeted active case-finding; and effective treatment of active disease (15). The available evidence suggests that screening for and treatment of LTBI may be a cost-effective intervention for population groups characterized by high prevalence of LTBI and/or high risk of progression to active TB (14).

Resource-limited countries should implement the existing WHO guidelines for people living with HIV and child contacts below 5 years of age (16).

### A social problem with medical repercussions

Tuberculosis is a disease essentially driven by social factors. These include poverty, crowded living conditions, malnutrition, drug and alcohol addiction, stigma, and social isolation. Nevertheless, for decades, since there were effective drugs available to treat TB, the approach to dealing with the disease has been mainly biomedical.

Poverty, however, does not only affect the patients who suffer from tuberculosis; it also shapes the TB programs that should be treating them. Frequently, control programs in high-burden countries lack trained health personnel, laboratory facilities, effective second-line drugs, and sufficient funds to provide optimal care for patients with drug-resistant TB.

If we are serious about eradicating TB, we must reconsider our current strategy and combine social components with biomedical interventions. This will require the development of alliances between government and civil society, as well as leadership and true political commitment at the highest level of government.

## CONCLUSIONS: MEASURES FOR FACING THE CHALLENGES

To confront the major problems mentioned above, there are four essential steps, in terms of funding, laboratory capacity, treatment adherence, and new affordable drugs.

### Adequate funding

National TB programs in low-income countries continue to rely on international donors for almost 90% of their financing. In 2016, investments in low- and middle-income countries fell almost US$ 2 billion short of the US$ 8.3 billion needed. In 2014, government expenditures on health were less than the WHO benchmark of at least 6% of gross domestic product (GDP) in150 countries. Drug-resistant tuberculosis in high-burden countries is consuming a significant proportion of the overall TB budget. The cost of treating a drug-susceptible case in 2014 ranged from US$ 100 to US$ 500 in most high-burden countries, whereas the cost of treating an MDR-TB patient typically ranged from US$ 5 000 to US$ 10 000 (4). A mathematical model suggests that almost two-thirds of MDR-TB cases are secondary to ongoing transmission, which is supported by molecular epidemiologic studies during MDR-TB outbreaks. This extensive transmission of MDR-TB is in part due to the diagnostic delay, thus stressing the urgent need for rapid diagnostics, comprehensive contact tracing, and effective treatment of drug-resistant cases. Implementation of these prevention and control measures must be a priority for policymakers in charge of controlling and eliminating tuberculosis (3).

### Enhanced laboratory capacity

Laboratory capacity urgently needs to be increased at a global level, especially in high-burden countries, in order to reach a larger number of TB patients and screen them for drug resistance at an earlier stage (17).

### Improved adherence to treatment

One of the main obstacles for TB control in most high-burden countries is a lack of treatment adherence, leading to irregular drug intake and the selection of drug-resistant mutations. The directly observed treatment, short-course-plus (DOTS-Plus) strategy needs to advance to broader, universal implementation, despite the overwhelming additional finances involved.

In its 2016 update of the treatment guidelines for drug-resistant tuberculosis (18), the WHO changed its recommendations for patients with rifampicin-resistant or multidrug-resistant TB who had not previously been treated with second-line drugs and in whom resistance to fluoroquinolones and second-line injectable agents had been excluded or was considered highly unlikely. For those persons, WHO recommended a shorter MDR-TB regimen, of 9 to 12 months, instead of a conventional regimen of 20 to 24 months.

However, that was a conditional recommendation, due to very low certainty in the evidence.

The STREAM trial is a multicenter randomized trial of non-inferiority design currently comparing short-course regimens to the treatment currently recommended by the WHO in patients with MDR pulmonary TB with no evidence of fluoroquinolone or kanamycin resistance (19). One of these short-course regimens is a fully oral nine-month regimen that includes bedaquiline, and another is a six-month regimen that also includes bedaquiline and a second-line injectable agent. The premise is that these short-course regimens (especially the fully oral regimen) will be as effective as the conventional regimen and will increase adherence to treatment.

### New *affordable* drugs

Only two new drugs have been developed for TB in the past 40 years, bedaquiline and delamanid (20, 21). Both are expensive and unaffordable for the national TB programs in most developing countries. In April 2015, the U.S. Agency for International Development (USAID), through the Stop TB Partnership, launched a bedaquiline donation program for the treatment of patients with drug-resistant TB. The program will provide 30 000 treatment courses of bedaquiline to patients in more than 100 countries over a period of four years (22). Although this represents much needed and welcomed help, it is simply not enough when the WHO is estimating there are 480 000 new cases of MDR-TB *every year*, and with 10% of them being XDR-TB cases.

### Disclaimer

The author holds sole responsibility for the views expressed in the manuscript, which may not necessarily reflect the opinion or policy of the *RPSP/PAJPH* or PAHO. In addition, the views and opinions expressed in this article are the author’s and are not an official position of the Regional Green Light Committees of the Americas (of which the author is the president).
